# Machine learning-enhanced modeling approach for optimally predicting household level food insecurity in Ethiopia during COVID-19

**DOI:** 10.1038/s41598-026-53425-3

**Published:** 2026-05-21

**Authors:** Henok Wariso Waqo, Gezahegn Mekonnen Woldemedihn, Yehenew Getachew Kifle, Frank Konietschke, Zeytu Gashaw Asfaw

**Affiliations:** 1 Department of Statistics, Hawassa University, Hawassa, Ethiopia; 2 Department of Epidemiology and Biostatistics, School of Public Health, Addis Ababa University, Addis Ababa, Ethiopia; 3 Institutes of Medical Biometry and Statistics, Faculty of Medicine and Medical Center, University of Freiburg, Freiburg, Germany; 4 Department of Mathematics and Statistics, University of Maryland Baltimore County, Baltimore, MD USA; 5 Institute of Medical Biometrics and Clinical Epidemiology, Charité Universitätsmedizin Berlin, Berlin, Germany

**Keywords:** Food insecurity, COVID-19, ML-models, Prediction, Regularization, Validation, Health care, Mathematics and computing, Medical research, Risk factors

## Abstract

Food insecurity remains a critical global challenge, with low-income countries such as Ethiopia bearing a disproportionate burden. In settings where frequent data collection is limited, developing predictive models provides a cost-effective means of anticipating risks to enhance rapid life-saving action, supporting timely evidence-based interventions. This study develops predictive models, applying Machine Learning (ML) approaches, capable of accurately forecasting household-level food insecurity. This study used data from the Ethiopia-High Frequency Phone Survey, collected by World Bank. The performances of Machine Learning models and classical logistic regression model, in predicting households’ food insecurity, were compared. Predictive models were trained and validated (internally and temporally) to evaluate model generalizability over time. ML models significantly outperformed the traditional logistic regression model in predicting household food insecurity. Based on the Brier score, the ML models demonstrated higher predictive accuracy and better calibration than the classical model. Regularization through optimized hyperparameters improved model stability and feature selection. While ridge, lasso, and elastic-net regressions produced similar coefficient directions, they differed in the number of selected predictors—the lasso model identified minimum key variables with comparable predictive accuracy. Temporal validation confirmed that the ML models maintained strong predictive performance, demonstrating their reusability and generalizability across time. The lasso regression model demonstrated strong predictive capability by selecting a manageable set of relevant features, resulting in reduced model complexity and improved interpretability. Integrating such ML models into food security monitoring systems can help policymakers design timely and data-driven interventions, enabling proactive responses in resource-constrained environment.

## Introduction

Food insecurity has increased worldwide since 2020, driven by the outbreak of COVID-19 disruptions, with Sub-Saharan Africa facing the greatest burden. Ethiopia, already strained by political unrest, climate shocks, and import dependency, experienced one of the world’s worst food crises in 2022, ranking 101 st among 125 countries on the Global Hunger Index^1–3^ .

Despite various programs (such as the Productive Safety Net Program, the World Food Programme, and several government initiatives) designed to alleviate hunger, the food insecurity crisis has consistently persisted, highlighting the need for actionable insights and improved prediction to support evidence-based interventions addressing this urgent challenge.

Studies on household food insecurity in Ethiopia have used statistical approaches ranging from logistic, Poisson, and linear regression to advanced multilevel, mixed-effects, spatial, Geo-additive mixed effect, and multistate Markov models^4–14^. These models enable the assessment of the determinant factors of food insecurity and capture spatial, temporal, spatio-temporal, and state-to-state variations, reflecting the various dimensions and complexity of the problem. However, they were based on a common applied researching approach focussing on how accurately parameters can be estimated without taking in to account the accuracy of prediction and validating the model’s reproducibility and transportability to forecast future food insecurity occurrences realistically.

Although both classical statistical models and Bayesian approaches used in previous studies can learn from data, they may face limitations in capturing complex nonlinear relationships, high-dimensional interactions, and feature selection compared to more flexible machine learning methods. These limitations may result in challenges such as reduced accuracy and limited generalizability, potentially leading to poorer predictive performance^15,16^.

A prediction model has a special concern in developing nations like Ethiopia, where obtaining realistic and recent datasets is rare. In contrast, the problem of food insecurity occurs more frequently in low-income countries compared to middle- and high-income countries. Additionally, repeatedly researching the food insecurity problem as it occurs is not feasible due to various constraints, making predictive models more cost-effective. Thus, in low-income settings, developing improved prediction models and validating their reusability to predict other datasets makes a vital contribution to alleviating households’ food insecurity and realizing Sustainable Development Goal 2.1, which aims to end hunger by 2030.

Despite its extensive application in various fields, from financial markets to disease epidemiology, predictive modeling remains scarce in previous food insecurity studies, leaving a critical gap. Optimizing prediction accuracy is a crucial concern for the effective implementation of strategies aimed at ensuring food security. Thus, the current study aims to address this gap by employing Machine Learning methods (ridge, lasso, and elastic net regression) that effectively select key quantified features of food insecurity, provide actionable and interpretable insights for policymakers, and improve prediction for more targeted and effective interventions.

Machine Learning (ML) is a popular approach that addresses these issues and outperforms in model prediction performance^16–21^.

Building on this rationale, we employed machine learning techniques to develop robust, data-driven prediction models, capable of accurately forecasting food insecurity under varying shock conditions. Therefore, this study aims to bridge the prediction gap by providing actionable insights to guide timely and targeted responses to food insecurity in Ethiopia, thereby transforming these responses from reactive measures into proactive, evidence-based actions.

## Methods

### Data source description and study design

The present study employed round 3 and round 6 data from the Ethiopia-High Frequency Phone Survey (EHFPS-HH) 2020–2023 dataset. The World Bank gathered the dataset in collaboration with Ethiopian Central Statistical Authority (CSA), aiming to provide realistic data that can be used in assessing social and socio-economic problems resulting from shocks, such as food insecurity, to design evidence-based policies. The study’s sampling design was based on the Ethiopian Socio-economic Survey (ESS), which is a representative sample of households in the country. Previous studies conducted on food insecurity in Ethiopia did not consider developing a predictive model with enhanced prediction capability and validating its reusability for accurately predicting in the subsequent time periods. To select the relevant features from the set of predictors applying ML methods, the present investigation used data from Round 3 and Round 6 because they include information on households’ food insecurity with the largest and most significant number of features (23 predictors) collected from a representative sample of 3051 and 2688 households, respectively. The former was used to develop a predictive model and internally validate it via splitting the data set into training (80%) and test set (20%). In contrast, the latter was used as a test dataset to temporally validate the developed model (by round 3 data) for ensuring its re-usability and generalizability in optimally predicting food insecurity in the subsequent periods.

### Study variables

#### Food security and its measurement

Following the comprehensive definition of FAO (FAO, 2000), food security is “a situation in which all people, at all times, have physical and economic access to sufficient, safe and nutritious food that meets their nutritional needs and food preferences for an active and healthy life.” Food security challenges can arise across the domains of *availability*, *access*, *utilization/nutrition*, and *stability* and can be studied at multiple levels (global, national, community, household, individual). Because including all dimensions in a single study is often infeasible, we primarily assess food insecurity in the *access* domain, consistent with the FAO and the Sustainable Development Goals (SDGs). A household is considered food insecure when it cannot reliably access the food required for a healthy, active, and dignified life. Despite the efforts made so far, there is no single instrument that fully captures every dimension of food security^22,23^. Consequently, measurement approaches vary across studies. In this work, we utilize the *Food Insecurity Experience Scale* (FIES), an eight–item experience-based scale designed for direct household assessment and aligned with the FAO, the Global Report on Food Crises, and the SDG monitoring framework^24–26^.

Let ${Y_{hj}}$ denote the response of household h to the ${j^{th}}{\mathrm{~~}}$ FIES item (j = 1, 2, …, 8), coded as:

$${Y_{hj}}=\left\{ {\begin{array}{*{20}{c}} {1,~~~~~~~~if~~household~h~exprienced~the~condition~in~item~j} \\ {0,~~~~~~~~~if~~household~h~did~not~exprience~the~condition~} \end{array}~} \right.$$

Under the FIES rule, a household is classified as *food insecure* if it reports at least one affirmative response across the eight items. Define the household-level indicator

1 $${H_h}={\rm I}(\sum\limits_{{j=1}}^{8} {{Y_{hj}}} \geqslant 1)$$

so that the food-insecurity status for household *h* satisfies

2 $${H_h}\sim Bernoulli(P)$$

where $p=\Pr ({H_h}=1)$ is the probability that a randomly selected household is food insecure.

#### Predictor variables

We consider the following household features as predictors of household’s food insecurity (variable codes in typewriter for clarity).

- Sex of household head (HH_gender); Age of household head (HH_age); Residence (HH_residence); Employment status (HH_employment); Receipt of assistance (HH_assistance); Income change since COVID-19 (HH_income).
- Non-farm business activity (HH_nonfarm); Farming activity (HH_farm); Access to financial services (HH_fin_service); Worry about COVID-19 (HH_worried_Covid19); Perceived financial threat from COVID-19 (HH_fin_threat).
- Sources of livelihood support since last call: farming/livestock/fishing (lc1_farm), business (lc1_bus), wage employment (lc1_wage), domestic remittances (lc1_rem_dom), foreign remittances (lc1_rem_foreign), income from properties, investments, and savings (lc1_isp), pension (lc1_pension), government support (lc1_gov), NGO charity support (lc1_ngo), and other sources (lc1_other).
- Handwashing with soap after being in public (Handwashing_freq); Mask wearing in public (Maskwearing_freq).

### Models

The present study employed ML and non-ML models to assess the models’ predictive performances and select the model that optimally predicts household-level food insecurity. From the current dataset, the food insecurity status of a household (study variable) is measured as dichotomous (food secure or food insecure). To compare a classical model prediction performance with ML models, we used logistic regression because it is commonly employed to model binary outcome variables and has been widely used in previous studies to model food insecurity.

Among machine learning modelling approaches, we used regularization methods (ridge, lasso, and elastic net) because the interpretation of penalized logistic regression results is relatively straightforward. Penalized regression models provide a suitable approach for quantifying key drivers of food insecurity, offering actionable and interpretable insights for policymakers that are essential for designing timely and targeted interventions in resource-constrained settings such as Ethiopia. Compared with other machine learning methods such as Random Forests, Neural Networks, and deep learning approaches, penalized regression models were preferred in this study due to their interpretability and ability to identify key predictors.

#### Non-ML model

##### Classical and Bayesian logistic regression (LR)

When we have a large number of features, the standard multivariable LR is expected to face the problem of over-fitting and multicollinearity. This leads to increased model complexity, increased residual variance, reduced model parsimony, and interpretability challenges^15,27^.

Given the LR model,

3 $${\pi _i}=\frac{{\exp ({\beta _0}+{\beta _1}{x_1}+{\beta _2}{x_2}+.....+{\beta _p}{x_p})}}{{1+\exp ({\beta _0}+{\beta _1}{x_1}+{\beta _2}{x_2}+.....+{\beta _p}{x_p})}}=\frac{{\exp ({\beta _i}^{T}X)}}{{1+\exp ({\beta _i}^{T}X)}}$$

where ${\beta _0}$ is the intercept and $\beta ={({\beta _1},{\beta _2}......,{\beta _p})^T}$ are regression parameters. Then, the likelihood of classical logistic regression is given by:

4 $$L(\beta ;{y_i},X)=\mathop \prod \limits_{i}^{n} {\pi _i}^{{yi}}{(1 - {\pi _i})^{(1 - yi)}}=\mathop \prod \limits_{i}^{n} \left\{ {{{(\frac{{\exp ({\beta _i}^{T}x)}}{{1+\exp ({\beta _i}^{T}x)}})}^{yi}}{{(1 - \frac{{\exp ({\beta _i}^{T}x)}}{{1+\exp ({\beta _i}^{T}x)}})}^{1 - yi}}} \right\}$$

The Bayesian logistic model’s likelihood derived from posterior distribution is:

5 $$\pi ({\beta _i}/data)=\mathop \prod \limits_{i}^{n} \left\{ {{{(\frac{{\exp ({\beta _i}^{T}x)}}{{1+\exp ({\beta _i}^{T}x)}})}^{yi}}{{(1 - \frac{{\exp ({\beta _i}^{T}x)}}{{1+\exp ({\beta _i}^{T}x)}})}^{1 - yi}}} \right\}*\prod\limits_{{i=1}}^{k} {\frac{1}{{\sqrt {2\pi {\sigma _i}^{2}} }}\exp } \left\{ { - \frac{1}{2}\left( {\frac{{{\beta _i} - {\mu _i}}}{{{\sigma _i}^{2}}}} \right)} \right\}$$

where ${\beta _i} \approx N({\mu _i},{\sigma _i}^{2})$ with prior distribution given by

6 $$P({\beta _i})=\frac{1}{{\sqrt {2\pi {\sigma _i}^{2}} }}\exp \left\{ { - \frac{1}{2}\left( {\frac{{{\beta _i} - {\mu _i}}}{{{\sigma _i}}}} \right)} \right\}$$

From the likelihoods of both classical and Bayesian estimations, it can be clearly seen that there is no hyper-parameter imposed to improve the prediction accuracy of the model for future data not used in parameter estimation^15,28^.

#### ML models (penalized regression)

Supervised ML, in particular penalized regression (ridge, lasso, and elastic net), shrinks the size of coefficients toward zero and retains relevant predictors, cancelling unnecessary features (in the case of LASSO and Elastic Net) to optimize the model’s prediction. The process imposes hyperparameters that tune extreme regression coefficients and select relevant features in a way that the variance is reduced at the cost of a negligible increase in bias up to the point where the minimum Test Mean Square Error (TMSE) is achieved. This leads to an optimal decision where loss is minimized, ensuring improved prediction and model validation for observations not used in model training^16,18,28^^,40^.

Letting *X* refers to features of a household’s food insecurity and *Y* refers to the household’s food insecurity (response), we consider supervised machine learning to find an optimal function $\hat {f}(X)$ that minimizes a loss$L(Y,\hat {f}(X))$. The process trades bias and variance by smoothing at the point of optimality.

**Ridge regression (L2 penalization)**

Ridge shrinks all regression coefficients toward smaller, but non-zero values, by imposing a penalty parameter $\lambda$ on the shrinkage term ${\left\| \beta \right\|_2}^{2}$. The estimator solves

7 $${\hat {\beta }_{Ridge}}=\arg {\hbox{min} _\beta }\left\{ {\sum\limits_{1}^{n} {({y_i}} - {\beta _0} - \sum\limits_{j}^{p} {{\beta _j}{x_{ij}}} {)^2}+\lambda \sum\limits_{1}^{p} {{\beta _j}^{2}} } \right\}$$

which is equivalent to minimizing the residual sum of squares subject to an *ℓ*2-ball constraint. Ridge is effective when many predictors have small, roughly equal effects and are correlated.

**Least Absolute Shrinkage and Selection Operator (LASSO; L1 regularization)**

LASSO shrinks coefficients and can set some values to zero, performing variable selection by imposing the penalty parameter *λ* on the shrinkage term${\left\| \beta \right\|_1}$:

8 $${\hat {\beta }_{Lasso}}=\arg {\hbox{min} _\beta }\left\{ {\sum\limits_{{j=1}}^{n} {({y_i}} - {\beta _0} - \sum\limits_{{j=1}}^{{}} {{\beta _j}{x_{ij}}} {)^2}+\lambda \sum\limits_{{j=1}}^{p} {\left| {{\beta _j}} \right|} } \right\}$$

LASSO is helpful when only a subset of predictors is truly influential.

**Elastic net regression (L1/L2 regularization)**

Elastic net combines ridge and lasso penalties to balance selection and shrinkage. With mixing parameter *α* ∈ [0, 1] and overall strength *λ >* 0:

9 $${\hat {\beta }_{Elastinet}}=\arg {\hbox{min} _\beta }\left\{ {\sum\limits_{{j=1}}^{n} {({y_i}} - {\beta _0} - {x_i}^{T}\beta {)^2}+\lambda \left\{ {\alpha \sum\limits_{{j=1}}^{p} {\left| {{\beta _j}} \right|+\frac{{1 - \alpha }}{2}\sum\limits_{{j=1}}^{p} {{\beta _j}^{2}} } } \right\}} \right\}$$

Setting *α* = 1 recovers LASSO; *α* = 0 recovers ridge. Elastic net is often preferred when predictors are grouped and highly correlated.

In practice, ridge can perform better when many predictors have effects of similar magnitude; lasso can be superior when only a few predictors are strongly influential. Cross-validation, sample splitting, and bootstrapping are commonly used to select penalties and compare the approaches^27,28^.

### Model validation

Validation strategies of the predictive models can be assessed through internal or external validation. Internal validation evaluates model performance on resampled or held-out data from the same development set using K-fold cross-validation, repeated CV, bootstrapping, or split sample schemes^18,28,29^.

External validation examines generalizability on independent data not used for model development. It can be temporal (same setting, later time period) or geographical (different setting/population). Temporal validation checks reproducibility on a later wave of the same population; geographical validation assesses transportability to new sites and populations^29–32^.

In this study, to ensure that the models are suitable for future food-insecurity risk prediction, we evaluate both internal (resampling-based) and external (temporal) validation procedures.

#### Internal validation

The predictive models developed were internally validated using Round-3 data via splitting– sample, such that 80% of the data were used for training (model fitting) and the remaining 20% for testing (validation). The penalty parameter *λ* that yielded the minimum prediction error was chosen. Among the 3051 households, 1842 were food insecure, showing an approximate class-imbalance ratio of 60:40. As a rule of thumb, class imbalance has to be mitigated using techniques such as *Synthetic Minority Oversampling Technique* (SMOTE) or using F1-score that compensates for the imbalance when the majority to minority ratio exceeds 65: 35. In the current study, the models were evaluated with balanced accuracy (F1_score) and discrimination metrics: Concordance index or Receiver Operating Characteristic (ROC)–AUC that balance the existing non-uniform distribution of class labels, and Brier-score metrics following Clark^27^; Ganapathy et al.^15^; Irfan et al.^18^; Dormosh et al.^33^; Tiruneh et al.^21^; Abebaw et al.^30^.

*Accuracy:* Accuracy is defined as the percentage of responses correctly classified from the confusion matrix. The accuracy of the model can be measured by the total accuracy, precision, recall, or F1–score. Total accuracy can be misleading under non-uniform class distributions, since it may over-represent the majority class. The F1_score, which provides a harmonic mean of precision and recall, used by the current study is defined as.

10 $${F_1}\_score=2x\frac{{\Pr ecisionX\operatorname{Re} all}}{{\Pr ecision+\operatorname{Re} all}}$$

where $$\Pr ecision=\frac{{TP}}{{TP+FP}}$$, and $$\operatorname{Re} call=\frac{{TP}}{{TP+FN}}$$.

The F1-score is a balanced measure that combines precision and recall, adjusting for class imbalance. A model with F1_score > 0.5 indicates predictive power above random chance^27,34^.

*Brier-score:* The Brier-score measures the mean-squared difference between predicted probabilities and observed outcomes.

11 $$Brier - Score=\frac{1}{n}\sum\limits_{{i=1}}^{n} {{{(pi - yi)}^2}=E\left[ {{{(1 - p)}^2}} \right]}$$

It ranges from 0 (perfect accuracy) to 1 (maximal inaccuracy); smaller values indicate better predictive performance.

*Concordance index/ROC-AUC.* The ROC curve plots the True-Positive Rate (sensitivity) against the False-Positive Rate (1 – specificity) for varying thresholds. The Area Under the Curve (AUC) ranges from 0 to 1; 0.5 represents random guessing, and 1 represents perfect classification. Higher AUC values reflect stronger discrimination. These complementary metrics provide a full assessment of model performance, mitigating bias from class imbalance.

The best-performing model is selected based on these internal validation metrics for potential use in future predictions of household food insecurity.

#### Temporal validation

Internal validation relies on data drawn from the same population and may not generalize beyond it. Temporal validation assesses the reproducibility of predictive performance using data collected at a later time from the same setting^29,30,33^. This addresses the main drawback of internal validation—the shared sampling source of training and testing data.

Temporal validation ensures that a model trained on one survey round can predict food-insecurity outcomes in subsequent rounds, demonstrating re-usability and robustness across time. Such testing enhances the model’s ability to capture time-varying risk and socio-economic dynamics^30,31,35^.

In the present study, temporal validation compared predictions based on Round-3 data with Round-6 data collected from the same population (with similar variables but different sampling times). The evaluated predictive models—using F1_score, ROC-AUC, and Brier-score—showed satisfactory reproducibility and transportability, confirming their suitability for future household food-insecurity risk prediction in related populations.

## Results

To address the aim of this study, we used the third- and sixth-round survey data from the Ethiopia-High Frequency Phone Survey (EHFPS–HH) conducted between 2020 and 2023.

Round-3 data were gathered from 3058 representative households, of which 99.77% provided complete responses, and only 0.23% had missing values occurring completely at random. Round-6 data were collected from 2704 households, from which 99.41% were complete, with a non-response rate of 0.59% also occurring completely at random. After cleaning for missing values (dropping seven rows from round-3 and six rows from round-6), the analysis was performed on 3051 households (round-3) for model development and internal validation, and on 2688 households (round-6) for temporal validation. Both ML and non-ML models were fitted using R software to evaluate and compare their predictive ability. The best-performing model was then validated to optimally predict household food-insecurity risk for targeted intervention^36^.

### Prediction performance of non-ML and ML models

In this section, we compare the predictive ability of three ML models—ridge, lasso, and elastic net regression—with one non-ML model, classical logistic regression (LR), in predicting household food insecurity. Table 1 summarizes the predictive performances of all models based on accuracy (F1_score), concordance index (ROC-AUC or C-statistic), and Brier-score.

The results indicate that all three ML models outperform logistic regression across all metrics. In particular, the ML models achieved superior Brier-score performance: ridge (0.0297), lasso (0.0314), and elastic net (0.0305), each reflecting improved probabilistic prediction accuracy and calibration, compared with logistic regression (0.2087). These results confirm that the penalized ML methods offer better generalization and accuracy than the standard logistic regression model for household food-insecurity prediction in the study population.

**Table 1:** Prediction performances of the fitted models using Round-3 data splitting.

Models employed	Accuracy (F1_score)	ROC (C-statistic)	Brier Score
Logistic regression	0.6856	0.7169	0.2087
Ridge regression	0.7815	0.7673	0.0298
Lasso regression	0.7837	0.7636	0.0314
Elastic net regression	0.7825	0.7619	0.0305

### Penalty parameters tuned to optimize the prediction of ML models

To obtain the optimal tuning parameter *λ*, we used 5-fold cross-validation with explicitly designed fold IDs (via cv.glmnet) so that ridge, lasso, and elastic-net shared identical folds for fair comparison. The penalty parameter governs the amount of shrinkage; it determines both the magnitude of the regression coefficients and, for lasso/elastic-net, the number of features retained in the final model.

**Fig. 1:**
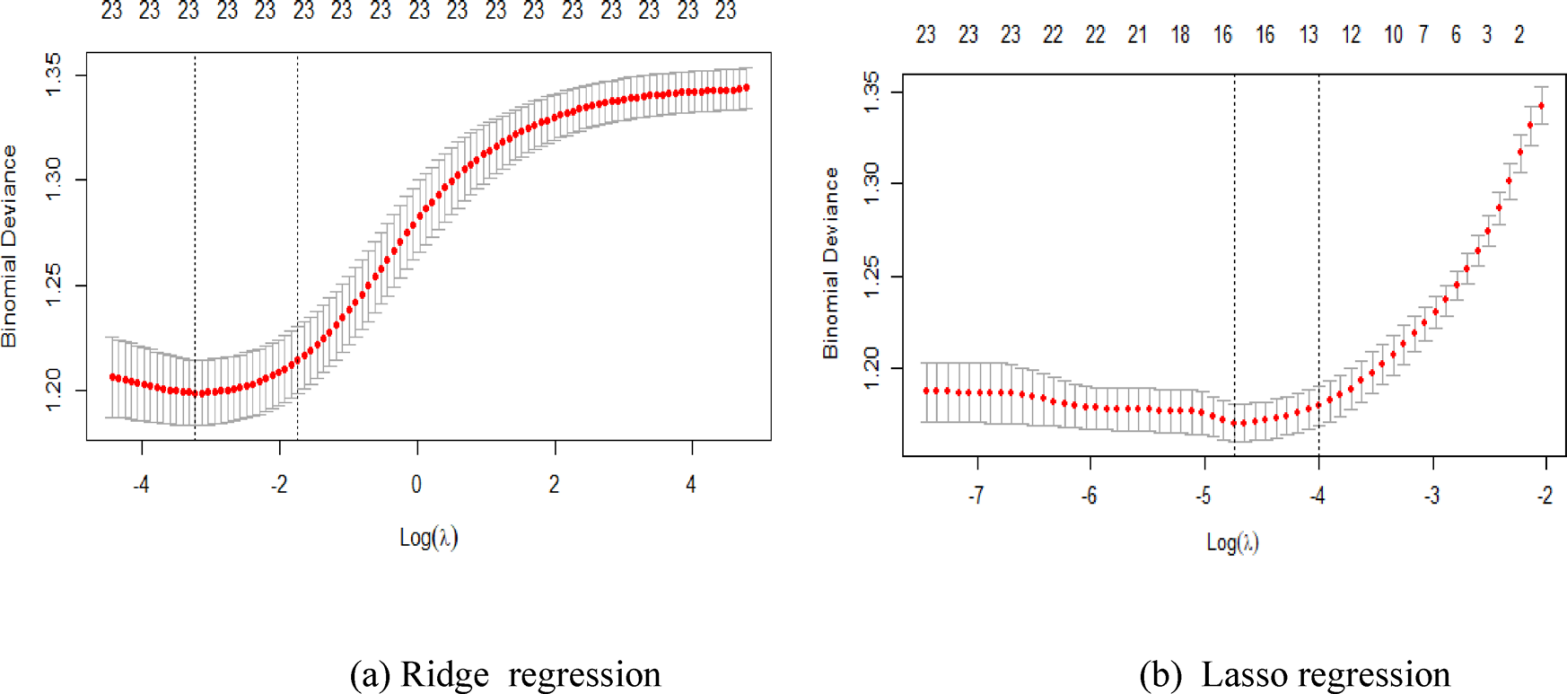
Cross-validated deviance vs. log (*λ*) with *λ*min and *λ*1se indicated by vertical dotted lines; numeric labels show non-zero coefficients.

**Fig. 2:**
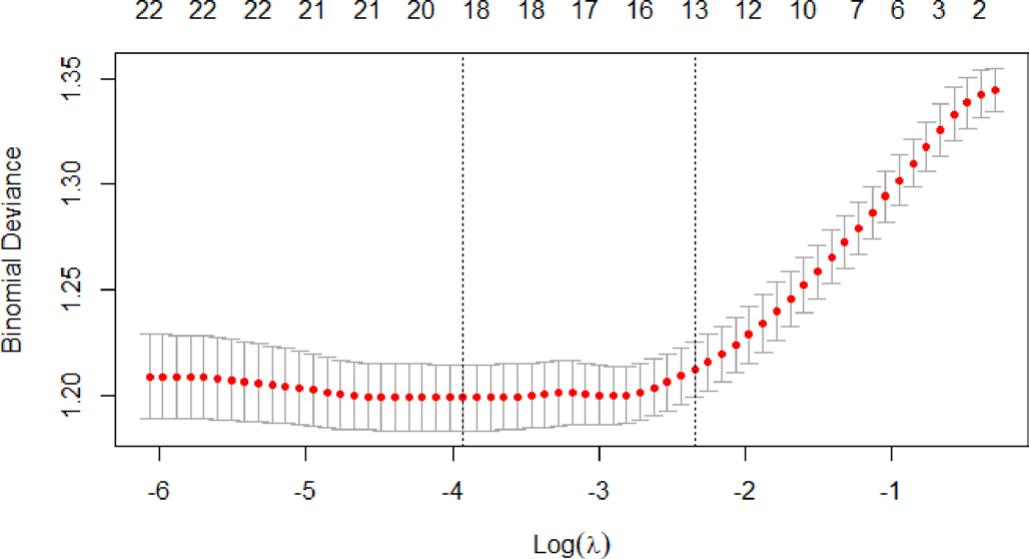
Elastic-net regression: cross-validated deviance vs. log (*λ*) with *λ*min and *λ*1se (vertical dotted lines).

Each cross-validation curve (Figs. 1a and 2) shows two special values: *λ*min (often written lambda.min), which gives the minimum mean cross-validated error, and *λ*1se (lambda.1se), which selects the most regularized model whose error is within one standard error of the minimum (vertical dotted lines).

For this study, we selected *λ*min for model fitting, as it yielded the lowest out-of sample error. The numbers above each curve indicate the count of non-zero coefficients at a given log (*λ*). In our fitted models, the number of retained predictors were 23 (ridge at $\lambda \hbox{min} ={\mathrm{-3}}{\mathrm{.2259}}$), 16 (lasso at $\lambda \hbox{min} ={\mathrm{-4}}{\mathrm{.8308}}$), and 18 (elastic-net at $\lambda \hbox{min} ={\mathrm{-3}}{\mathrm{.9285}}$), respectively.

### Results on estimates of ML models’ coefficients

The results in Table 2 indicate that the number of features selected and the magnitudes of the corresponding regression coefficients differ across the ridge, lasso, and elastic-net regression models, although the coefficient signs are largely consistent. Ridge regression selected all 23 features as predictors, whereas lasso and elastic-net regression retained 18 and 16 features, respectively, as notable contributors to household food insecurity, shrinking the others to zero.

Considering the direction of the coefficients in the three penalized regression models, the household head’s sex and income, government support since the last call, other sources of support, and household’s mask-wearing frequency were positively associated with predicted outcome of food insecurity. In practical terms, this implies that households headed by females, those who experienced income loss, those who received government support, and those who wore masks less frequently tended to face higher risks of food insecurity prediction.

Conversely, households headed by older individuals, living in urban areas, are being employed, receiving assistance during COVID-19, engaging in non-farm business, Ploughing their farm, being less worried about COVID-19, facing fewer financial threats, and accessing financial services were inversely associated with food insecurity. Moreover, households that benefited from wage employment, pensions, NGOs, and remittances (from domestic or abroad), business activity, agricultural production, or income from investments and savings were inversely related to the predicted outcomes of food insecurity.

**Fig. 3:**
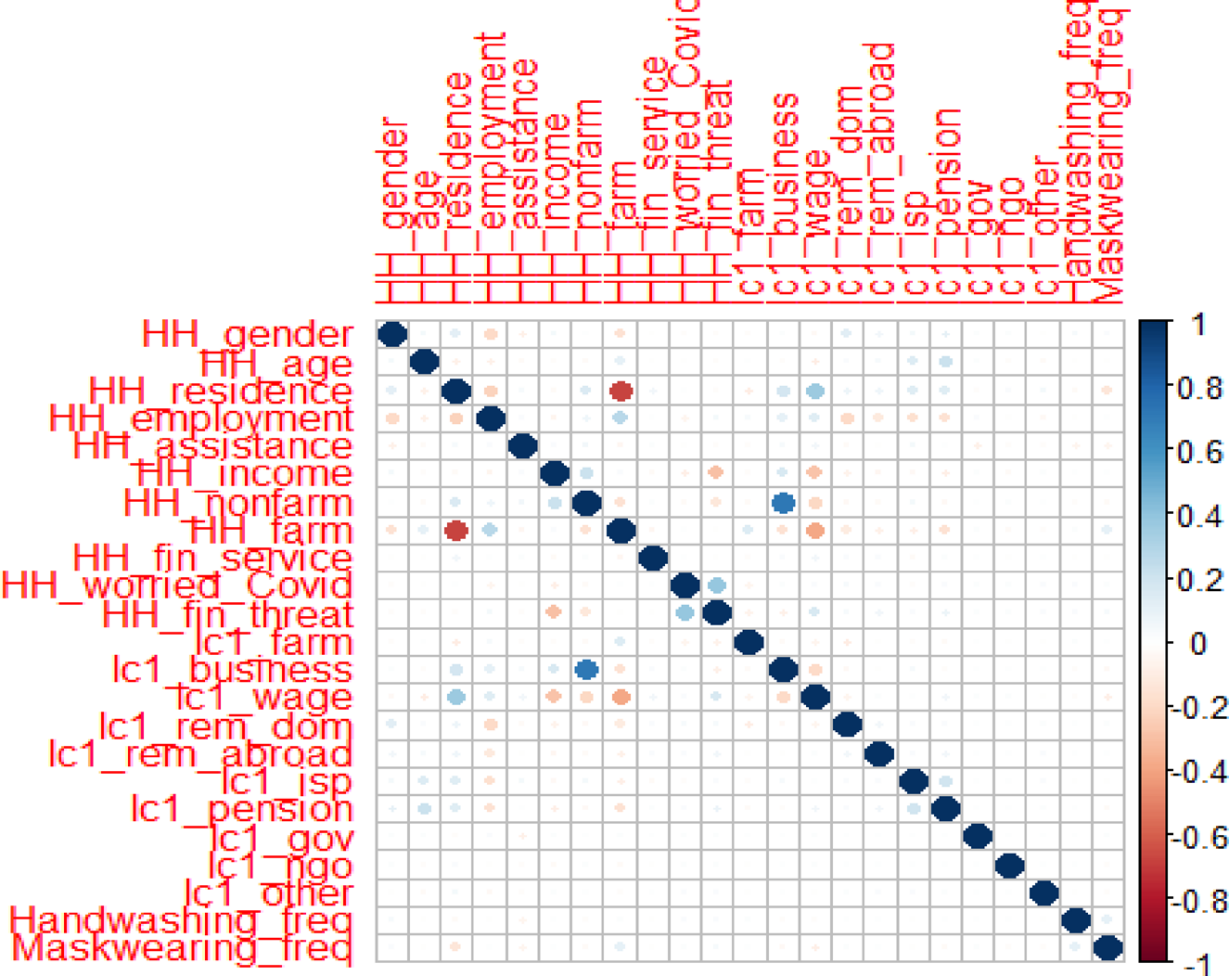
Visual plot for detecting automatic variable reordering and hidden patterns among predictors.

Among the three models, lasso regression yielded the most parsimonious solution by shrinking the coefficients of six variables—household employment status, farm ploughing, business, domestic remittances, NGO support, and hand-washing frequency—to zero. To investigate why theoretically important variables (e.g., employment status) were excluded, we used a visual correlation analysis via the corrplot () function in R, which automatically reorders variables to highlight hidden correlations and interaction patterns. The resulting plot (Fig. 3) shows several inter-relationships: employment is moderately correlated with multiple predictors; business support is positively related to non-farm business ownership; and ploughing the household’s farm is inversely correlated with urban residence and wage employment. These findings suggest that feature collinearity and interdependence may explain the automatic exclusion of some variables by the Lasso and Elastic-Net models.

Despite these eliminations, the focus of this study is on predictive performance rather than individual effect interpretation. Penalized regression techniques such as ridge, lasso, and elastic-net enable the identification of key predictors while improving model parsimony and predictive accuracy in assessing household food insecurity risk.

**Table 2:** Estimates of penalized regression coefficients.

Variables with possible responses	Ridge regression	Lasso regression	Elastic net regression
(Intercept)	1.310962749	1.109644061	1.22186157
HH_gender (1 = Male; 2 = Female)	0.300514237	0.263137001	0.29895556
HH_age (1 = below 40; 2 = 40 a7 above)	−0.057140514	-	−0.03567497
HH_residence (1 = Rural; 2 = Urban)	−0.241107689	−0.205759414	−0.22630614
HH_employment (0 = No, 1 = Yes)	−0.020055072	-	-
HH_income (1 = Not reduces, 2 = Reduced)	0.615141549	0.649730377	0.64663075
HH_assistance (0 = No, 1 = Yes)	−0.666192629	−0.636611055	−0.68029077
HH_nonfarm_bus (0 = No, 1 = Yes)	−0.468606711	−0.541629459	−0.53158649
HH_farm (0 = No, 1 = Yes)	−0.006199939	-	-
Financial_service (0 = No, 1 = Yes, received the service, 2. Yes, did not receive the service)	−0.032244580	−0.017739543	−0.02663667
HH_worried (1 = Very worried, 2 = Somewhat worried, 3 = Not too worried, 4 = not worried at all)	−0.116597585	−0.094428143	−0.11006508
HH_Fin_threat (1 = Substantial, 2 = Moderate, 3 = Not much, 4 = Not at all)	−0.295963749	−0.316982780	−0.31159849
lc1_farm_LF (0 = No, 1 = Yes)	0.304293175	0.305512462	0.30860407
lc1_business (0 = No, 1 = Yes)	−0.077724228	-	−0.03133577
lc1_wage (0 = No, 1 = Yes)	−0.433658167	−0.437953162	−0.44597376
lc1_rem_domestic (0 = No, 1 = Yes)	−0.015864638	-	-
lc1_rem_foreign (0 = No, 1 = Yes)	−0.365667876	−0.074266201	−0.26191339
lc1_isp (0 = No, 1 = Yes)	−0.699672231	−0.730346374	−0.73299834
lc1_pension (0 = No, 1 = Yes)	−0.281371969	−0.179549924	−0.24213442
lc1_government (0 = No, 1 = Yes)	0.029058726	0.009521495	0.02087609
lc1_ngo (0 = No, 1 = Yes)	−0.010849678	-	-
lc1_other (0 = No, 1 = Yes)	0.871271587	0.503621366	0.77739510
Hand_washing (1 = Always, 2 = Mostly, 3 = Half, 4 = Sometimes, 5 = None, 6 = Not public places)	−0.007177725	-	-
Mask_wearing (1 = Always, 2 = Mostly, 3 = Half, 4 = Sometimes, 5 = None, 6 = Not public places)	0.027292104	0.019252935	0.02503143

### Coefficient paths of penalized regression

The coefficient paths illustrate the magnitude and direction of estimated coefficients as a function of the regularization parameter *λ* for each penalized regression model. These paths provide insight into how model shrinkage affects variable inclusion and stability across different *λ* values.

**Fig. 4:**
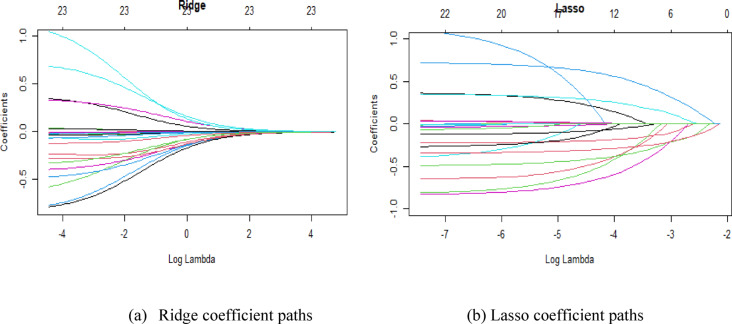
Coefficient paths of penalized regression models showing the estimated regression coefficients across log(*λ*) values.

For ridge regression (Fig. 4a), all lines representing estimated regression coefficients cross the vertical line drawn at $\lambda \hbox{min}$ of ridge i.e. $\lambda ={\mathrm{-3}}{\mathrm{.2259}}$(in log scale). This indicates that ridge regression included all 23 variables, while shrinking them toward smaller magnitudes.

In contrast, for lasso regression (Fig. 4b), only 16 lines cross the vertical line corresponding to lasso$\lambda \hbox{min} ={\mathrm{-4}}{\mathrm{.8308}}$. This shows that lasso model selected 16 predictors as the key determinants of household food insecurity, while shrinking seven noisy or weak predictors to zero. Thus, the lasso model achieves a more parsimonious representation by retaining only the most influential features while discarding redundant ones.

### Results on validating the prediction model

#### Results on internal validation

In this sub-section, following the result in part 3.1 that ML models (ridge, lasso, and elastic-net) showed the improved food insecurity prediction, their internal validity is evaluated. The Brier-score values (Table 1) were 0.0298, 0.0314, and 0.0305 for ridge, lasso, and elastic-net regression, respectively. These indicate that the models’ probability predictions are very close to the true outcomes, demonstrating well-calibrated predictions. Thus, the penalized regression models exhibit strong predictive performance for assessing the future risk of household food insecurity.

The models’ predictive accuracy, measured by the F1_score, was 0.7815 for ridge, 0.7837 for lasso, and 0.7825 for elastic-net regression. These results show that the models achieved strong balance between Precision and Recall, revealing that they performed well in correctly identifying positive cases while keeping false positives reasonably low.

**Fig. 5:**
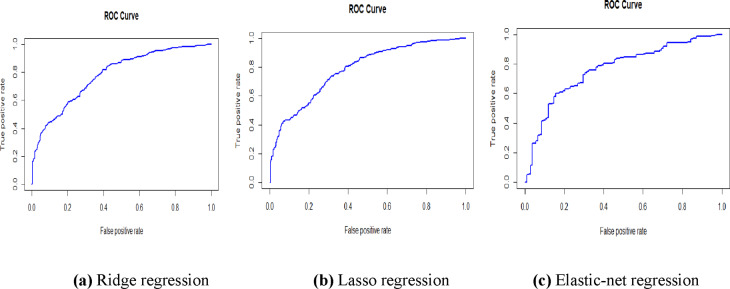
ROC curves of penalized regression models showing the discriminative performance of ridge, lasso, and elastic-net regression based on internal validation data.

The concordance indices (C-statistics) for the ridge, lasso, and elastic-net models were 0.7673, 0.7636, and 0.7619, respectively, confirming that the models correctly discriminated more than 76% of the predictions. The Receiver Operating Characteristic (ROC) curves for the three models are displayed in Fig. 5a and c. These plots show that when the fitted models are used for prediction, more than 76% of the households predicted as food insecure are indeed observed as food insecure. The area under the ROC curves further demonstrates strong discriminative performance and supports the reliability of the fitted models for future food-insecurity prediction.

##### Results on temporal validation

As shown in Table 3, temporal validation on Round-6 data confirms robust performance for all three penalized models. The Brier scores (0.0342–0.0371) indicate that the models’ predicted probabilities are, on average, very close to the true outcomes, supporting good overall model accuracy and calibration under temporal shift. The F1_score ranges from 0.6824 to 0.6923 on the temporally distinct test set, indicating a good balance between precision and recall. These results suggest the models maintain reasonable predictive performance under temporal shift.

Discrimination remains solid: ROC C-statistics between 0.6957 and 0.7081 showing that each model correctly ranks ≈ 70% of the pairs, substantially above chance (0.5). Collectively, these results indicate the models generalize reasonably well across survey rounds and are suitable for forward prediction of household food-insecurity risk in the related population.

**Table 3:** Prediction performance of the predictive model using Round-6 data as test data set.

ML-models to be validated	Accuracy (F1_score)	ROC (C-statistic)	Brier Score
Ridge regression	0.6923	0.7081	0.0342
Lasso regression	0.6824	0.7005	0.0349
Elastic net regression	0.6896	0.6957	0.0371

## Discussions

The current study focused on developing a predictive model that can be used for optimal prediction of households’ food insecurity and validating it for accurately detecting the future risk of such occasions. Most previous studies on food insecurity mainly emphasized assessing the prevalence, variations, determinants, and other dynamics of the problem^2,6–8,13,14^, but not on improving the predictive ability of the models. Predictive modeling, which plays a vital role in making accurate projections of food insecurity, has not been adequately considered by previous studies. It is therefore deemed crucial, especially for resource-limited countries such as Ethiopia, where food insecurity is frequent and severe.

Based on this rationale, the present study was initiated to develop a predictive model capable of accurately forecasting food insecurity in various shock manifestations and to validate it internally and temporally to ensure reproducibility and generalizability. The developed models were evaluated using data collected in multiple rounds (EHFPS-HH 2020–2023), thereby ensuring the robustness of the prediction framework^29,30,33,35^. The findings are instrumental in delivering cost-effective, reusable, and optimally performing models, advancing the science of food insecurity prediction.

###  Performance comparison between ML and non-ML models

The results revealed that the machine learning (ML) models significantly outperformed the non-ML logistic regression model across all predictive performance measures. In contrast to other studies^4–6,11^, this study found that the penalized regression models—ridge, lasso, and elastic-net—substantially improved the prediction of food insecurity under varying shock conditions. On the other hand, the finding of the present study is consistent with studies^15–18,21,37^. The superior performance of ML models lies in their estimation techniques that minimize total mean squared error (MSE) through regularization, effectively reducing over fitting and multicollinearity while optimizing predictive accuracy.

### Interpretation and feature selection

To propose the optimal predictive model for future use, the study considered both predictive performance metrics and interpretability. Despite near-equal performance across all ML models, the lasso regression model was identified as the most parsimonious and interpretable, owing to its ability to select manageable and relevant predictors. This result aligns with the principle of model simplicity and parsimony^16,28,38^^,40^ which supports using fewer but meaningful predictors for interpretable and transferable prediction frameworks.

Similar to Verdonk et al.^39^, Greenwood et al.^16^, & James et al.^28^, in the lasso and elastic-net models, seven and five predictors, respectively, had coefficients shrunk to zero, representing sparsity in the presence of multicollinearity among variables or irrelevance in predicting the outcome. Predictors such as household employment status and farm ploughing were zeroed out, likely due to high correlation with related variables such as business ownership or residence. Thus, these variables may be expressed as functions of others, as supported by the correlation visualization in Fig. 3.

For lasso regression, the coefficients for the age of the household head and business support—variables correlated with non-farm business—were shrunk to zero, consistent with Verdonk et al.^39^, and Sievering et al.^20^, who noted that regularized ML models incorporate interactions and non-linearities among correlated variables. Likewise, domestic remittances, hand washing frequency, and NGO support during COVID-19 were also reduced to zero in both lasso and elastic-net regression models due to the limited independent contribution to the outcome.

### Temporal validation and model generalizability

The study’s temporal validation demonstrated consistent predictive power across rounds, confirming re-usability over time. The Brier score results in temporal validation (0.034–0.037) indicated that all ML models demonstrated high prediction accuracy and good calibration, closely matching internal validation results. Although the F1_scores and ROC values were slightly lower than those in internal validation, this decline is expected due to population and time differences between the development and validation datasets.

The findings align with Dormosh et al.^33^, showing that predictive models tend to perform slightly better within-sample than out-of-sample. Nonetheless, the models maintained strong discrimination (C-statistics ≈ 0.70), suggesting that the developed models can accurately predict food insecurity using later survey data from the same population, as confirmed by Abebaw et al.^30^, and Lopes et al.^35^.

### Strengths and limitations

This study has several notable strengths. First, it used a realistic, nationwide dataset (EHFPS-HH 2020–2023) developed by major international organizations (World Bank, USAID, CSA, WBG, and GFF), ensuring data quality and representativeness. Second, it focused on Ethiopia—a low-income country where food insecurity prediction research is limited although it frequently faces a serious hunger—contributing valuable evidence. Third, both internal and temporal validation were conducted, confirming reproducibility and robustness.

However, the study has limitations. It employed only cross-sectional data (Round-3 and Round-6), limiting longitudinal inference. Future research should apply advanced ML techniques such as Random Forest (RF), Gradient Boosting (XGB), Neural Networks (NN), and Deep Learning (DL), which explore more complex nonlinear interactions (Gholami et al., 2022; Busker et al., 2024), to longitudinal datasets with larger sample sizes and additional covariates (e.g., climatic, agricultural, and market variables) to further improve prediction reliability. Finally, although this study primarily addressed low-income settings, future research should expand to middle- and high-income contexts to assess the geographical validity of the predictive framework. Collecting consistent data across diverse regions will be essential for refining a globally adaptable model for food insecurity risk prediction.

## Conclusions

In the present study, all machine learning models—ridge, lasso, and elastic-net demonstrated superior predictive performance compared to the classical logistic regression model, which has been widely applied in previous studies on food insecurity. The penalized regression models provided reliable and trustworthy predictive accuracy, with the lasso regression model achieving comparable performance while retaining the smallest number of predictors. This highlights the suitability of the lasso regression model for future applications in predicting food insecurity, as it achieves an optimal balance between model complexity, interpretability, and parsimony.

Temporal validation of the developed predictive models confirmed their reusability and consistent predictive accuracy across different time periods. This implies that the models can be effectively used to predict persistent household food insecurity of similar population in subsequent time periods.

To enhance forecast precision and support evidence-based decision making, the use of regularized ML models, particularly lasso regression, is recommended for policy institutions, government agencies, and development partners. These models can facilitate the timely identification of at-risk households and inform the design of targeted, data-driven interventions to mitigate recurring challenges of food insecurity.

## Data Availability

All data are fully available without restriction and can be accessed from the database: [https://microdata.worldbank.org/index.php/catalog/3716/get-microdata](https:/microdata.worldbank.org/index.php/catalog/3716/get-microdata).

## Abbreviations

AUC: Urea Under a Curve
COVID-19: Coronavirus Disease of 2019
FAO: Food and Agricultural Organization
FIES: Food Insecurity Experience Scale
EHFPS: Ethiopia-High Frequency Phone Survey
LASSO: Least Absolute Shrinkage and Selection Operator
LR: Logistic Regression
ML: Machine Learning
ROC: Receiver Operating Characteristic
WB: World Bank
WHO: World Health Organization

## References

[1] FAO. Food security and nutrition in the world. In *Safeguarding against economic slowdowns and downturns. Rome, FAO. Licence*: (Vol. 10, Issue 9). (2023). 10.1016/S2213-8587(22)00220-0

[2] Akalu & Wang. *Does the female-headed household suffer more than the male-headed from Covid-19 impact on food security? Evidence from Ethiopia* (2023). 10.1016/j.jafr.2023.100563 PMC10041883 37016627

[3] Bout & Hunger, G. *Global Hunger Index 2023: Nigeria* (2023).

[4] Alemu, Z. A., Ahmed, A. A., Yalew, A. W. & Simanie, B. Spatial variations of household food insecurity in East Gojjam Zone, Amhara Region, Ethiopia: Implications for agroecosystem-based interventions. *Agric. Food Secur.* **6** (1), 1–9. 10.1186/s40066-017-0113-9 (2017).

[5] Belayneh, M., Loha, E. & Lindtjørn, B. Seasonal variation of household food insecurity and household dietary diversity on wasting and stunting among young children in a drought prone area in South Ethiopia: A cohort study. *Ecol. Food Nutr.* **00**(00), 1–26. 10.1080/03670244.2020.1789865 (2020). 10.1080/03670244.2020.1789865 32672490

[6] Ayele, A. W., Kassa, M., Fentahun, Y. & Edmealem, H. Prevalence and associated factors for rural households food insecurity in selected districts of East Gojjam Zone, Northern Ethiopia: Cross-sectional study. *BMC Public Health* **20**(1), 1–14. 10.1186/s12889-020-8220-0 (2020). 32033552 10.1186/s12889-020-8220-0 PMC7007667

[7] Belachew Sime, E. Determinants of household food insecurity in rural Ethiopia: Multiple linear regression (classical and bayesian approaches). *Int. J. Theor. Appl. Math.* **6**(5), 64. 10.11648/j.ijtam.20200605.12 (2020).

[8] Dessie, Z. G., Zewotir, T. & North, D. The spatial modification effect of predictors on household level food insecurity in Ethiopia. *Sci. Rep.* **12** (1), 1–11. 10.1038/s41598-022-23918-y (2022). 36369275 10.1038/s41598-022-23918-y PMC9652367

[9] Garratt, E. Food insecurity in Europe: Who is at risk, and how successful are social benefits in protecting against food insecurity?. *J. Soc. Policy* **49**(4), 785–809. 10.1017/S0047279419000746 (2020).

[10] Gebrie, Y. F. Bayesian regression model with application to a study of food insecurity in household level: A cross sectional study. *BMC Public Health* **21**(1), 1–10. 10.1186/s12889-021-10674-3 (2021). 33785006 10.1186/s12889-021-10674-3 PMC8008667

[11] Niles, M. T. & Salerno, J. D. A cross-country analysis of climate shocks and smallholder food insecurity. *PLoS. One* **13**(2), 1–14. 10.1371/journal.pone.0192928 (2018). 10.1371/journal.pone.0192928 PMC5825057 29474383

[12] Roba, K. T. et al. Seasonal variations in household food insecurity and dietary diversity and their association with maternal and child nutritional status in rural Ethiopia. *Food Secur.* **11**(3), 651–664. 10.1007/s12571-019-00920-3 (2019).

[13] Rudin-Rush, L., Michler, J. D., Josephson, A. & Bloem, J. R. Food insecurity during the first year of the COVID-19 pandemic in four African countries. *Food Policy* **111**(February), 102306. 10.1016/j.foodpol.2022.102306 (2022). 35783573 10.1016/j.foodpol.2022.102306 PMC9234064

[14] Wubetie, H. T., Zewotir, T., Mitku, A. A. & Dessie, Z. G. Heliyon spatiotemporal modeling of household ’ s food insecurity levels in Ethiopia. *Heliyon* **10**(12), e32958. 10.1016/j.heliyon.2024.e32958 (2024). 39668987 10.1016/j.heliyon.2024.e32958 PMC11637179

[15] Ganapathy, S., Harichandrakumar, K. T., Penumadu, P., Tamilarasu, K. & Nair, N. S. Comparison of Bayesian, Frequentist and machine learning models for predicting the two-year mortality of patients diagnosed with squamous cell carcinoma of the oral cavity. *Clin. Epidemiol. Glob. Health* **17**(February), 101145. 10.1016/j.cegh.2022.101145 (2022).

[16] Greenwood, C. J. et al. A comparison of penalised regression methods for informing the selection of predictive markers. *PLoS One* **15**(11 November), 1–14. 10.1371/journal.pone.0242730 (2020). 10.1371/journal.pone.0242730 PMC7678959 33216811

[17] Fenta, H. M., Zewotir, T. & Muluneh, E. K. A machine learning classifier approach for identifying the determinants of under-five child undernutrition in Ethiopian administrative zones. *BMC Med. Inform. Decis. Mak.* **21**(1), 1–12. 10.1186/s12911-021-01652-1 (2021). 34689769 10.1186/s12911-021-01652-1 PMC8542294

[18] Irfan, S., Meerza, A., Imran, S., Meerza, A. & Ahamed, A. *Food Insecurity Through Machine Learning Lens: Identifying Vulnerable Households Food Insecurity Through Machine Learning Lens : Identifying Vulnerable Authors : University of Louisville*. (2021). 10.22004/ag.econ.314072

[19] Qasrawi, R. et al. Machine learning approach for predicting the impact of food insecurity on nutrient consumption and malnutrition in children aged 6 months to 5 years. *Children* **11**(7), 1–16. 10.3390/children11070810 (2024). 10.3390/children11070810 PMC11274836 39062259

[20] Sievering, A. W. et al. Comparison of machine learning methods with logistic regression analysis in creating predictive models for risk of critical in-hospital events in COVID-19 patients on hospital admission. *BMC Med. Inform. Decis. Mak.* **22**(1), 1–14. 10.1186/s12911-022-02057-4 (2022). 36437469 10.1186/s12911-022-02057-4 PMC9702742

[21] Tiruneh, S. A., Vu, T. T. T., Rolnik, D. L., Teede, H. J. & Enticott, J. Machine learning algorithms versus classical regression models in pre-eclampsia prediction: A systematic review. *Curr. Hypertens. Rep.* **26**(7), 309–323. 10.1007/s11906-024-01297-1 (2024). 38806766 10.1007/s11906-024-01297-1 PMC11199280

[22] Maxwell, D., Coates, J. & Vaitla, B. How do different indicators of household food security compare ? Empirical evidence from Tigray. *Feinstein International Center* (2013).

[23] Upton, J. B., Cissé, J. D. & Barrett, C. B. Food security as resilience: Reconciling definition and measurement. *Agric. Econ. (U.K.)* **47**(February 2015), 135–147. 10.1111/agec.12305 (2016).

[24] Adjognon, G. S., Bloem, J. R. & Sanoh, A. The coronavirus pandemic and food security: Evidence from Mali. *Food Policy* **101**(September 2020), 102050. 10.1016/j.foodpol.2021.102050 (2021). 36570061 10.1016/j.foodpol.2021.102050 PMC9758592

[25] Cafiero, C., Viviani, S. & Nord, M. Food security measurement in a global context: The food insecurity experience scale. *Meas. J. Int. Meas. Confed.* **116**(November), 146–152. 10.1016/j.measurement.2017.10.065 (2018).

[26] Smith, M. D., Rabbitt, M. P. & Coleman- Jensen, A. Who are the World’s food insecure? New evidence from the Food and Agriculture Organization’s Food Insecurity Experience Scale. *World Dev.* **93**(January 2017), 402–412. 10.1016/j.worlddev.2017.01.006 (2017).

[27] Clark, J. S. Model Assessment and Selection. In *Models for Ecological Data*. (2020). 10.2307/j.ctv15r5dgv.9

[28] James, G., Witten, D., Hastie, T. & Tibshirani, R. *Multiple Testing*. (2021). 10.1007/978-1-0716-1418-1_13

[29] Ramspek, C. L., Jager, K. J., Dekker, F. W. & Zoccali, C. External validation of prognostic models: What, why, how, when and where?. *Clin. Kidney J.* **14**(1), 49–58. 10.1093/ckj/sfaa188 (2021). 33564405 10.1093/ckj/sfaa188 PMC7857818

[30] Abebaw, S., Lorber, D., Teede, H. & Enticott, J. Temporal validation of machine learning models for pre-eclampsia prediction using routinely collected maternal characteristics: A validation study temporal validation of machine learning models for pre-eclampsia prediction using routinely collected mater. *Comput. Biol. Med.* **191**(May), 110183. 10.1016/j.compbiomed.2025.110183 (2025). 40228443 10.1016/j.compbiomed.2025.110183

[31] Debray, T. P. A. et al. Original articles a new framework to enhance the interpretation of external validation studies of clinical prediction models. *J. Clin. Epidemiol.* **68**(3), 279–289. 10.1016/j.jclinepi.2014.06.018 (2015). 25179855 10.1016/j.jclinepi.2014.06.018

[32] Gallitto, G. et al. *External validation of machine learning models - registered models and adaptive sample splitting Background The anatomy of a prospective predictive modelling study,* 1–12 (2023).

[33] Dormosh, N. et al. External validation of a prediction model for falls in older people based on electronic health records in primary care. *J. Am. Med. Dir. Assoc.* **23**(10), 1691-1697e.e3. 10.1016/j.jamda.2022.07.002 (2022). 35963283 10.1016/j.jamda.2022.07.002

[34] Villacis, A. H., Badruddoza, S. & Mishra, A. K. A machine learning-based exploration of resilience and food security. *Appl. Econ. Perspect. Policy.* **46**(4), 1479–1505. 10.1002/aepp.13475 (2024).

[35] Lopes, R. R. et al. Temporal validation of 30-day mortality prediction models for transcatheter aortic valve implantation using statistical process control – An observational study in a national population. *Heliyon*10.1016/j.heliyon.2023.e17139 (2023). 37484279 10.1016/j.heliyon.2023.e17139 PMC10361331

[36] Fransiska, H., Soleh, A. M. & Notodiputro, K. A. *Evaluation of machine learning models based on household food insecurity data in Indonesia* (2025).

[37] Feffer, M., Kapoor, P. & Dodt, S. *Predicting food insecurity*. (2021).

[38] Breiman, L. Statistical modeling: The two cultures. *Stat. Sci.* **16** (3), 199–215. 10.1214/ss/1009213726 (2001).

[39] Verdonk, C., Verdonk, F. & Dreyfus, G. How machine learning could be used in clinical practice during an epidemic. *Crit. Care*. **24** (1), 1–3. 10.1186/s13054-020-02962-y (2020). 32456690 10.1186/s13054-020-02962-y PMC7250254

[40] James, G., Witten, D., Hastie, T. & Tibshirani, R. *Springer Texts in Statistics An Introduction to Statistical Learning* (n.d.).

